# Assessing Hospital Readmission Risk Factors in Heart Failure Patients Enrolled in a Telemonitoring Program

**DOI:** 10.1155/2013/305819

**Published:** 2013-04-27

**Authors:** Adrian H. Zai, Jeremiah G. Ronquillo, Regina Nieves, Henry C. Chueh, Joseph C. Kvedar, Kamal Jethwani

**Affiliations:** ^1^Laboratory of Computer Science, Massachusetts General Hospital of Harvard Medical School, Boston, MA 02114, USA; ^2^Partners Center for Connected Health, Partners Healthcare, Boston, MA 02114, USA; ^3^Department of Dermatology, Massachusetts General Hospital of Harvard Medical School, Boston, MA 02114, USA

## Abstract

The purpose of this study was to validate a previously developed heart failure readmission predictive algorithm based on psychosocial factors, develop a new model based on patient-reported symptoms from a telemonitoring program, and assess the impact of weight fluctuations and other factors on hospital readmission. Clinical, demographic, and telemonitoring data was collected from 100 patients enrolled in the Partners Connected Cardiac Care Program between July 2008 and November 2011. 38% of study participants were readmitted to the hospital within 30 days. Ten different heart-failure-related symptoms were reported 17,389 times, with the top three contributing approximately 50% of the volume. The psychosocial readmission model yielded an AUC of 0.67, along with sensitivity 0.87, specificity 0.32, positive predictive value 0.44, and negative predictive value 0.8 at a cutoff value of 0.30. In summary, hospital readmission models based on psychosocial characteristics, standardized changes in weight, or patient-reported symptoms can be developed and validated in heart failure patients participating in an institutional telemonitoring program. However, more robust models will need to be developed that use a comprehensive set of factors in order to have a significant impact on population health.

## 1. Introduction

Several predictive models can identify the risk status of patients with heart failure [1]. However, predictors used in those models are often not actionable, as they are typically based on demographic (e.g., age, race/ethnicity) or clinical data (e.g., medical history, billing or laboratory data).

In our previous work, we aimed to identify a subset of high-risk patients with reversible risk factors, as our goal was to prevent their readmission by connecting those patients to appropriate interventions. Since psychosocial factors might be a root cause for cardiac decompensation, we set ourselves to develop a multivariable logistic regression model based on psychosocial predictors [2]. In that work, we identified 5 psychosocial predictors “dementia,” “depression,” “adherence,” “declining/refusal of services,” and “missed clinical appointments” as significant predictors of readmission [2].

Similarly, patient-reported symptoms and other factors collected by a telemonitoring system could potentially serve as reversible predictors to eventually strengthen our original model. In fact, body weight gain among heart failure patients is already a known factor linked to early readmissions [3].

Telemonitoring is a promising innovation that allows clinicians to monitor patients with heart failure remotely for signs of clinical deterioration, enabling timely and effective intervention by the physician. There are a range of technologies that collect and transmit real-time patient data such as physical symptoms, blood pressure, weight changes, and electrocardiogram readings to a central location for evaluation [4, 5]. By monitoring symptoms that reflect a patient's physiological struggle to remain euvolemic, telemonitoring holds promise in reducing hospital readmissions and significantly improves patient morbidity and mortality, as well as quality of life [6]. A recent meta-analysis found that telemonitoring of heart failure patients reduced both all-cause mortality and heart failure-related hospitalizations by 44% and 21%, respectively [6, 7]. At the same time, other studies highlight that telemonitoring has no positive impact on readmissions, suggesting that the benefits of telemonitoring are not universally observed [7]. As a result, telemonitoring remains a costly and limited resource, which hospitals must utilize carefully and responsibly. It will thus be increasingly critical to be able to identify scenarios where telemonitoring contributes to improved clinical outcomes for different populations of patients.

Telemonitoring systems are producing increasing amounts of information that require the use of sophisticated algorithms to process, filter, and prioritize [8]. Going forward, these algorithms will play a key role in integrating data from multiple sources, evaluating a patient's clinical condition, and identifying patients at increased risk for hospitalization [8–10].

In summary, we had 3 goals in mind with this study. First, we aimed to validate the heart failure readmission model based on psychosocial factors we previously developed. This step was necessary to ensure that our previous model was applicable to the study population. Second, we set forth to develop a new predictive model based on patient-reported symptoms from a telemonitoring program. Finally, we planned to assess the impact of weight fluctuations on hospital readmission (weight fluctuation was used as a proxy to weight gain secondary to hypervolemia because our telemonitoring program intervened on weight gain).

## 2. Methods

The Partners Connected Cardiac Care Program (CCCP) is a joint telemonitoring effort between Partners Center for Connected Health and Partners Home Care designed to help patients better manage their heart failure and avoid rehospitalization. Enrolled patients are provided with Bosch's VitalNet suite of devices, consisting of a weight scale, blood pressure cuff, and pulse oximeter, to send their data and symptom information to the VitalNet portal via telephone line every day, at the same time each day, where telemonitoring nurses view the data and follow up accordingly. Failure to upload would generate a reminder phone call to the patients by the telemonitoring nurses. If patients uploaded data outside parameters, nurses would follow standing orders given by the cardiologists, or if necessary, send the cardiology team a clinical message. We selected 100 patients enrolled in the CCCP who completed the 4-month program between July 2008 and November 2011 following a hospitalization. The enrolled population included English-speaking men and women over the age of 18 years who were patients from Massachusetts General Hospital (MGH) with a diagnosis of heart failure. Using patient names and medical record numbers, relevant telemonitoring, readmission, and clinical data was extracted from the VitalNet portal data warehouse, as well as from the MGH Longitudinal Medical Record (LMR) electronic medical record system. Telemonitoring data recorded from the VitalNet portal included daily weight, blood pressure, pulse, O_2_ saturation, and severity of symptoms for each patient. We also recorded whether patients received a reminder call for their data, or any other clinical intervention by the nursing staff.

A previously developed hospital readmission prediction model using psychosocial factors [2] was validated on the patient population in the current study. A receiver operating characteristic (ROC) curve was generated for a range of probability threshold values, with readmission status for each patient based on whether the calculated readmission probabilities exceeded a given threshold value. A cutpoint of 0.3 was used to determine the model's impact on selecting and enrolling patients into the CCCP, and additional calculations were performed for sensitivity, specificity, positive predictive value, negative predictive value, and area under the ROC curve (AUC). The probability of readmission within 30 days of index admission was separately modeled with logistic regression using ten candidate predictors representing physical symptoms or factors reported by heart failure patients: shortness of breath, cough, pain, dizziness, tired, rapid heart rate, swollen ankles and feet, swollen abdomen, diet, and missed medications.

An “adjusted weight standard deviation” was used to assess the association of weight fluctuations with patient readmission risk, calculated as the standard deviation of all weights for each patient during the 4-month enrollment period adjusted by the mean weight. Two-sample *t*-tests were used to evaluate potential differences in adjusted weight standard deviation by readmission status. The multivariable logistic regression model included all predictors initially, and a *P* value of <0.05 was considered to be statistically significant. Statistical analyses were performed using SPSS PASW version 18 (SPSS Inc., Chicago, IL, USA). The Massachusetts General Hospital Institutional Review Board approved this study.

## 3. Results

### 3.1. Telemonitoring Population Characteristics

A total of 100 patients enrolled in the Connected Cardiac Care Program participated in the study, average age is 66.8 ± 13.6 years old with an adjusted weight standard deviation of 1.86 ± 1.24 lbs, and 38% readmitted to the hospital within 30 days for heart failure. There were approximately 17389 entries recorded over the study period for ten different heart-failure-related symptoms (Table 1). The top three reported symptoms (tiredness, pain, and swollen ankles and feet) contributed approximately 50% of the total volume of positive symptoms.

### 3.2. Using CCCP Patient Population for Validating Psychosocial-Factor Readmission Predictive Model

**Figure 1:**
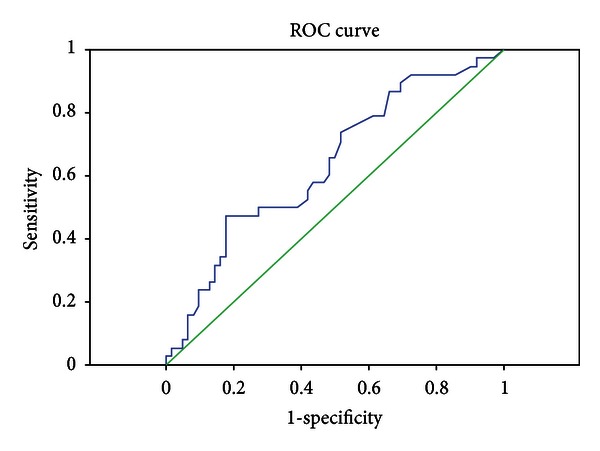
Receiver operating characteristic (ROC) curve validating psychosocial predictive model [2] on CCCP patient population.

Validation of the previously developed psychosocial factor-based predictive model for hospital readmissions was performed on the CCCP patient population, resulting in the receiver operating characteristics (ROC) curve detailed below (Figure 1). Furthermore, application of the prediction model to the CCCP patient population yielded an area under the curve (AUC) of 0.637 (95% CI 0.525–0.749), similar to the AUC of 0.670 from the original study [2]. Using a predefined probability threshold of 0.3, the sensitivity and specificity were found to be 0.87 and 0.32, respectively. The positive predictive and negative predictive values were calculated to be 0.44 and 0.8, respectively, at this defined cutpoint.

In order to assess the impact of using the predictive model as part of the criteria to enroll patients in the CCCP, we evaluated the potential reduction in patients readmitted for heart failure at varying cutpoints, as well as the fraction of patients who should have been readmitted but were missed (Table 2). Transitioning from a cutpoint of 0.28 to 0.30 leads to a 7% net reduction in the volume of readmitted patients, with approximately 3.3% of readmitted patients missed. Increasing the cutpoint 0.02 units further to 0.32 led to a 20% reduction in patient readmission volume and an 8.8% increased false negative rate. The results thus suggest that the predictive model was capable of identifying heart failure telemonitoring patients at higher risk for readmission along with showing greater reductions in readmitted patient volume at higher probability thresholds. However, this comes at the cost of higher false negative rates where more high-risk patients would not receive the care they needed.

### 3.3. Association of Telemonitoring Reported Heart Failure Symptoms with 30-Day Hospital Readmission Risk

Development of a logistic regression model based on ten different heart-failure-related symptoms reported by patients in the study showed that two predictors made small but statistically significant contributions in the final multivariable model (Table 3). More specifically, controlling for other heart failure symptoms, a patient reporting rapid heart rate was 1.062 times more likely to be readmitted within 30 days than a patient without a reported rapid heart rate (*P* < 0.05). Second, controlling for other physical symptoms and factors, patients who reported a swollen abdomen were at 0.970 decreased risk of 30-day hospital readmission for heart failure than those not reporting abdominal swelling (*P* < 0.05). Two covariates suggested a trend towards significance, with patients who reported swollen feet at potentially increased risk of readmission, while reports of missed medications were associated with a decrease in 30-day hospital readmission risk. No significant associations were detected for cough, dizziness, pain, diet, tiredness, and shortness of breath (*P* > 0.05 for all). With 4 covariates—rapid heart rate, swollen abdomen, swollen feet, and missed medication—combined, the telemonitoring-based readmission model yielded an AUC of 0.21, along with a sensitivity of 0.5, a specificity of 0.81, a positive predictive value of 0.61, and a negative predictive value of 0.72 at a cutoff value of 0.35.

### 3.4. Subanalysis of the Impact of Weight Fluctuations on Hospital Readmission Risk

**Figure 2:**
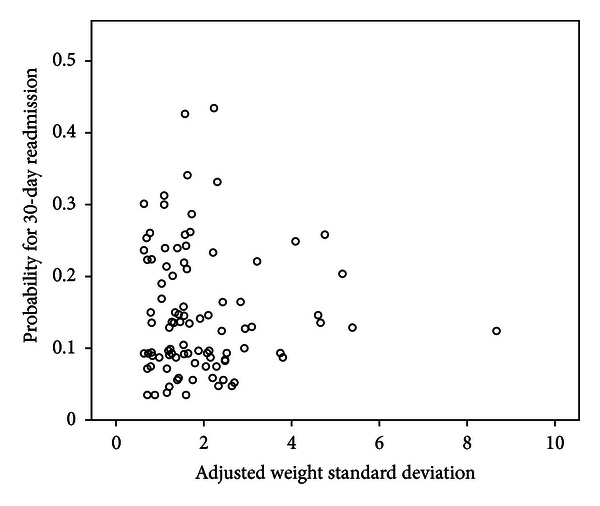
Scatterplot of 30-day readmission risk versus “adjusted weight standard deviation” for each patient.

When stratified by admission status, there was no difference between the “adjusted weight standard deviation” of patients readmitted to the hospital within 30 days for heart failure compared to those who were not readmitted (2.05 ± 1.53 versus 1.74 ± 1.03 lbs, *P* = 0.133). Using the physical symptoms reported by CCCP patients as a starting point, a regression model using 30-day readmission risk as a continuous outcome (not shown) was developed, and readmission risk was graphed as a function of standardized changes in weight for each patient (Figure 2). No correlation was found between the frequency of reported weight changes and likelihood of 30-day hospital readmission for heart failure (*P* = 0.83). In summary, no significant association between fluctuations in weight and risk for hospital readmission for heart failure could be detected in the telemonitoring patient population studied.

## 4. Discussion

The development of prediction models to identify patients at increased risk for hospital readmission can be a useful way of describing and prioritizing the severity of a patient's condition, particularly for those in telemonitoring programs for heart failure [4]. This study describes a systematic process of integrating data acquired daily through telemonitoring in order to validate existing algorithms or develop new ones applicable to this important patient population.

Our results showed a wide variety of positive symptoms reported by patients in the study. The top five symptoms included such factors as fatigue (tiredness) and shortness of breath, which have been known to be strongly associated with congestive heart failure [11]. Other findings can also be found in the literature and highlight the importance and utility of patient-reported symptoms in models to assess patient status [12]. By understanding which factors are commonly reported by patients, the most relevant subset of covariates can be selected even within the context of highly constrained prediction model development.

The validation of a previously developed model on patients in the CCCP—a completely different population than the earlier study but with similar clinical needs—highlights the importance of developing models that can be applied to new populations. While the AUC of the validated algorithm suggested only moderate discriminatory power, it was actually comparable to several other published prediction algorithms [2, 13, 14].

Additional studies support the development of real-time clinical prediction models capable of risk-stratifying patients along multiple dimensions, including patient-centric factors and the utilization of health services [15]. The use of patient-reported symptoms as potential predictors may provide an additional dimension to our psychosocial model for assessing readmission risk for heart failure patients enrolled in telemonitoring programs.

To achieve this goal, we developed an independent telemonitoring model based on patient-reported symptoms. There were two main reasons why we decided to develop a predictive model based on patient-reported symptoms. First, we wanted to find out whether variables based on patient-reported symptoms are significant predictors of early readmission in and of themselves. Second, we suspected that certain symptoms might be precursors of poor psychosocial behaviors for heart failure patients.

The telemonitoring model identified two symptoms that, while statistically significant, only marginally changed the odds of readmission. The results of our study are thus likely not sophisticated enough in their current form to associate patient-reported symptoms and early heart failure readmissions. Interestingly, the telemonitoring model with a AUC of 0.21 identified heart failure patients who are less likely to get readmitted. One likely explanation is that the telemonitoring program was intervening appropriately on patients reporting symptoms, thereby minimizing their likelihood for readmission.

In addition to patient-reported symptoms, we were also interested in assessing if weight fluctuations could serve as a proxy to weight gain to predict readmission. Unfortunately, fluctuations in weight were not strongly associated with readmission risk. The results of our study are thus likely not sophisticated enough in their current form to be used as the sole criteria for enrolling patients into telemonitoring. However, our findings support the idea that the trigger for hospital readmissions among heart failure patients originates from multiple, complex factors both intrinsic and extrinsic to the patient, and current algorithms that use only a portion of these variables may not suffice. Telemonitoring has the potential to prolong life and improve the quality of care, especially when combined with transitional care shown to decrease the risk of hospital readmission [6, 16, 17]. A more comprehensive predictive model for readmission should integrate the psychosocial and patient-reported characteristics described in our study, clinical and demographic factors, and relevant data about hospital operations [15]. Our goal is to eventually develop such a model.

## 5. Limitations

There were several limitations to our study. First, validation of an existing predictive model and development of new prediction models using a small number of patients in a single telemonitoring program may not be generalizable, and more comprehensive studies will be needed. Second, all patients in the study were enrolled in the CCCP by design, and thus all were being treated by physicians actively trying to minimize readmissions, which may have contributed to the low discriminatory ability of the prediction models. Finally, our study focused on a specific telemonitoring technology that may not be available in other institutions. However, the data collected and used to develop these models are likely commonly collected by numerous telemonitoring systems or available from related systems. Regardless, we believe that our use of a well-described patient population with a single, clear telemonitoring modality provides a good approach to developing more robust prediction algorithms in the future.

## 6. Conclusion

In summary, hospital readmission models based on psychosocial characteristics, standardized changes in weight, or patient-reported symptoms can be developed and validated in heart failure patients participating in an institutional telemonitoring program. However, more robust models will need to be developed that use a comprehensive set of factors (clinical, behavioral, psychosocial, and operational) in order to have a significant impact on population health.

## Figures and Tables

**Table 1:** Positive symptoms reported for the 100 patients enrolled in CCCP.

Symptoms	Frequency (% of total)
Tiredness	3817 (22.0)
Pain	2954 (17.0)
Swollen ankles and feet	1935 (11.1)
Diet	1839 (10.6)
Shortness of breath	1773 (10.2)
Cough	1547 (8.9)
Swollen abdomen	1481 (8.5)
Dizziness	1371 (7.9)
Rapid heart rate	627 (3.6)
Missed medications	45 (0.3)

**Table 2:** Impact of using psychosocial factor-based readmission predictive model at varying probability cutpoints.

Probability threshold (cutpoint)	% Reduction in readmitted patient volume	% Readmitted patients missed (false negatives)
0.28	18.0%	16.7%
0.30	25.0%	20.0%
0.32	45.0%	28.8%

**Table 3:** Multivariable heart failure symptom predictors of 30-day hospital readmission (*n* = 100).

Predictor	OR	95% CI	*P*
Missed medications	0.493	0.235–1.036	0.062
Cough	1.004	0.986–1.022	0.655
Dizziness	1.018	0.991–1.046	0.199
Pain	0.993	0.979–1.007	0.342
Diet	1.019	0.998–1.040	0.072
Rapid heart rate*	1.062	1.001–1.127	0.047
Shortness of breath	0.998	0.980–1.015	0.785
Swollen abdomen*	0.970	0.942–0.999	0.042
Swollen feet	1.022	0.999–1.045	0.06
Tiredness	1.000	0.987–1.012	0.966

*Indicates statistical significance *P* < 0.05.
